# Emerging roles of long non-coding RNAs in osteosarcoma

**DOI:** 10.3389/fmolb.2024.1327459

**Published:** 2024-03-07

**Authors:** Xun Liao, Rong Wei, Junxiu Zhou, Ke Wu, Jiao Li

**Affiliations:** ^1^ Frontiers Medical Center, Tianfu Jincheng Laboratory, Chengdu, Sichuan, China; ^2^ Laboratory of Molecular Oncology, Frontiers Science Center for Disease-related Molecular Network, State Key Laboratory of Biotherapy and Cancer Center, West China Hospital, Sichuan University, Chengdu, China

**Keywords:** osteosarcoma, long non-coding RNAs, signaling pathways, drug resistance, biomarkers

## Abstract

Osteosarcoma (OS) is a highly aggressive and lethal malignant bone tumor that primarily afflicts children, adolescents, and young adults. However, the molecular mechanisms underlying OS pathogenesis remain obscure. Mounting evidence implicates dysregulated long non-coding RNAs (lncRNAs) in tumorigenesis and progression. These lncRNAs play a pivotal role in modulating gene expression at diverse epigenetic, transcriptional, and post-transcriptional levels. Uncovering the roles of aberrant lncRNAs would provide new insights into OS pathogenesis and novel tools for its early diagnosis and treatment. In this review, we summarize the significance of lncRNAs in controlling signaling pathways implicated in OS development, including the Wnt/β-catenin, PI3K/AKT/mTOR, NF-κB, Notch, Hippo, and HIF-1α. Moreover, we discuss the multifaceted contributions of lncRNAs to drug resistance in OS, as well as their potential to serve as biomarkers and therapeutic targets. This review aims to encourage further research into lncRNA field and the development of more effective therapeutic strategies for patients with OS.

## 1 Introduction

Osteosarcoma (OS) is a highly prevalent and aggressive bone tumor that affects individuals across various age groups, including children, adolescents, and adults over 60 years of age (Mirabello et al., 2009). It is typically located in the metaphysis of the long bones, most commonly in the knee (50%), humerus (15%), or pelvic girdle (12%) (Bielack et al., 2002). Approximately 80% of patients initially present with a localized form of OS and no distant metastases (Bacci et al., 1997). Currently, the primary treatment modalities for OS include surgical resection, chemotherapy, and radiotherapy. Advances in surgical techniques and neoadjuvant chemotherapy have resulted in a 65%–70% cure rate when using cisplatin (DDP), doxorubicin (DOX) and methotrexate (MTX) as adjuvant chemotherapies (Whelan and Davis, 2018). However, the overall survival rate for individuals with distant metastases or multi-drug resistance (MDR) remains below 20% and has not shown significant improvement over an extended period of time (Bacci et al., 1997; Sakamoto and Iwamoto, 2008). The pathogenesis of OS is believed to originate from the malignant transformation of mesenchymal cells of an undefined stage of differentiation into osteoblasts (Klein and Siegal, 2006). Furthermore, numerous genetic alterations have been identified in OS, including dysregulation of p53 and Rb tumor suppressor genes, aneuploidies, and extensive disruption in the regulation of chromosomal arrangement, hinting at a possible primary defect in the DNA repair mechanisms. However, the detailed molecular mechanisms of OS remain unclear. Therefore, unraveling the mechanisms driving oncogenesis, progression and chemoresistance in OS, as well as identifying novel biomarkers for alternative therapies or improved efficacy of existing treatments, is of paramount importance in order to improve clinical outcomes.

Previous studies of OS primarily focused on protein-coding genes due to their importance in biological regulation, however, analysis of the human genome has revealed that less than 2% of its sequence is composed of protein-coding genes, while the remaining 98% is actively transcribed into non-coding RNAs (ncRNAs), previously considered as transcriptional “noise” (Rosenbloom et al., 2012). Recent evidence, however, suggests that these evolutionarily conserved ncRNAs have a significant role in a range of physiological and pathological processes (Huarte, 2015; Chi et al., 2019). lncRNAs are non-coding transcripts longer than 200 nucleotides and can be classified into sense, antisense, bidirectional, intronic, and intergenic transcripts (Ma et al., 2013; Mattick et al., 2023). To date, over 50,000 genes have been identified to generate lncRNAs, with tissue-, lineage-, and spatiotemporal-specific patterns (Iyer et al., 2015). Certain lncRNAs, such as HOTAIR, MALAT1 and H19, have been implicated in OS oncogenesis and progression by modulating signaling pathways (Wang B. et al., 2015; Li E. et al., 2017; Li H. et al., 2023). Furthermore, lncRNAs are associated with the response of OS patients to chemotherapy (Hahne and Valeri, 2018). Intriguingly, some lncRNAs are specific to OS and can be detected in body fluids, making them attractive biomarkers in liquid biopsy (Cai et al., 2017; Han et al., 2017; Yang Q. et al., 2019). For example, alkaline phosphatase (ALP) has displayed both good specificity and sensitivity when used for the diagnosis of OS(Levine and Rosenberg, 1979), however, serum HNF1A-AS1 is more effective than ALP in distinguishing OS from healthy individuals (Cai et al., 2017). Furthermore, pre-clinical studies have already demonstrated therapeutic targeting of lncRNAs, such as MALAT1 and HOXB-AS3 in cancer (Arun et al., 2016; Papaioannou et al., 2019), suggesting their potential clinical value in cancer diagnosis and treatment. In this review, we discuss the dysregulation and regulatory role of lncRNAs in the oncogenesis, progression, as well as chemoresistance of OS. Finally, we discuss the diagnostic and therapeutic potential of lncRNAs in OS.

## 2 Regulatory roles of lncRNAs on gene expression in cancers

**FIGURE 1:**
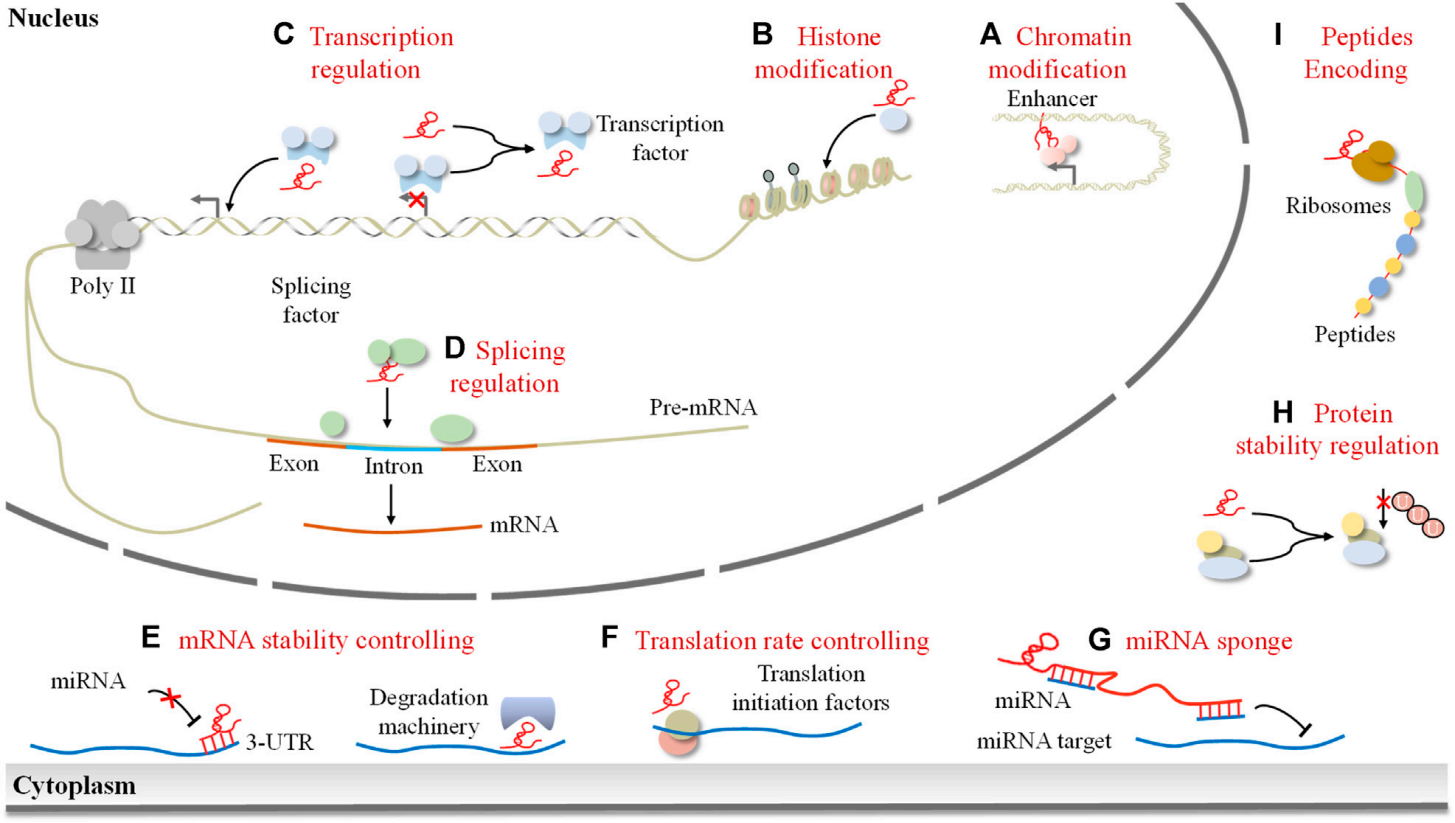
The mode of lncRNAs in the regulation of gene expression. LncRNAs exert their effects depending on their subcellular localization. In the nucleus, lncRNAs can control the epigenetic state of genes by **(A)** modifying chromatin architecture and **(B)** mediating chromatin/histone modifications, participating in the transcriptional process by **(C)** guiding transcription factors to induce or inhibit gene transcription and **(D)** regulating splicing of pre-mRNAs. Cytoplasmic lncRNAs can **(E)** regulate mRNA stability, **(F)** control translation rate, **(G)** act as a miRNA sponge, **(H)** mediate protein stability, and **(I)** encode peptides.

LncRNAs have emerged as crucial regulators of gene expression in cancer, exerting their influence through diverse mechanisms. Depending on their subcellular localization, lncRNAs perform distinct functions within the nucleus and cytoplasm. In the nucleus, lncRNAs orchestrate epigenetic modifications by recruiting chromatin remodeling and modification complexes (Figure 1A, B). They also collaborate with transcription factors to act as transcriptional regulators, modulating the expression of neighboring messenger RNAs (Figure 1C). Moreover, lncRNAs can interact with splicing factors or pre-mRNA molecules themselves, thereby influencing the splicing process (Figure 1D). Furthermore, lncRNAs participate in post-transcriptional gene regulation, impacting mRNA stability, translation, protein stability, and competing endogenous RNA (ceRNA) networks (Figures 1E–G). Intriguingly, studies have revealed that certain lncRNAs can encode peptides (Figure 1H). Collectively, lncRNAs are implicated in gene expression regulation at multiple levels.

### 2.1 LncRNAs in epigenetic regulation

LncRNAs are involved in epigenetic regulation, which can influence gene expression in a heritable manner without changing the underlying DNA sequence (Xiao et al., 2018). These enigmatic molecules have been unveiled as influential regulators, recruiting esteemed chromatin-modifying enzymes such as histone acetyltransferases (HATs), histone deacetylases (HDACs), and histone methyltransferases (HMTs). Through their interactions, these enzymes shape the expression patterns of transcription factors and their coregulators, thereby exerting a profound impact on gene expression. For example, the lncRNA HOTAIR guides a chromatin modification complex towards target genes, altering chromatin state and modulating their transcription (Tsai et al., 2010). HOTTIP, in turn, interacts with WDR5 through chromatin looping, enabling the WDR5-MLL complex to target HOXA gene promoters, promoting histone H3K4 trimethylation (Luo et al., 2019). LncRNAs also regulate DNA methylation through modulation of DNMT expression. TARID binds GADD45α, inducing demethylation of 5-methylcytosine on the TCF21 promoter, activating transcription, and suppressing tumor formation (Arab et al., 2014).

### 2.2 LncRNAs in transcriptional regulation

LncRNAs have been shown to regulate gene transcription through both cis- and trans-regulation. In cis-regulation, lncRNAs can act as transcriptional regulators by binding to regulatory sequences in the promoter region of their target genes, thus modulating the transcription of those genes. For instance, lncRNAs can recruit SWI/SNF complexes to the promoter of target genes, such as TCF7, to modulate the expression of these genes and consequently activate the Wnt signaling pathway, thus promoting the self-renewal of liver cancer stem cells (CSCs) and tumor proliferation (Wang Y. et al., 2015). On the other hand, in trans-regulation, lncRNAs can act as scaffolds, recruiting transcription factors and chromatin modifiers to regulate gene expression. For example, lncRNA AGAP2-AS1 has been demonstrated to recruit the enhancers of zeste homolog 2 (EZH2) and LSD1 to the promoter regions of KLF2 and large tumor suppressor 2 (LATS2), thus suppressing the transcription of KLF2 and LATS2 and promoting the progression of non-small cell lung cancer (Li W. et al., 2016). Additionally, lncRNA GAS5 has been found to fold into a DNA-like structure that binds to the glucocorticoid receptor (GR) and inhibits its transcription activity (Mourtada-Maarabouni and Williams, 2013).

### 2.3 LncRNAs in post-transcriptional regulation

At the post-transcriptional level, lncRNAs interact with molecules to modulate mRNA splicing, stability, protein stability, and subcellular localization (Yoon et al., 2013). They can influence mRNA translation as stabilizers, destabilizers, ribosomal recruiters, or mRNA decoys. Antisense lncRNAs regulate pre-mRNA alternative splicing, alone or with splicing factors. For example, lnc-Spry1 binds U2 small nuclear ribonucleoprotein auxiliary factor 65 kD, controlling variable splicing of fibroblast growth factor receptor pre-mRNA during the epithelial-mesenchymal transition (EMT) (Rodríguez-Mateo et al., 2017). LncRNAs also affect mRNA stability through their 3′UTR. For instance, lncRNA PXN-AS1-L binds the 3′UTR of Paxillin (PXN) mRNA, preventing miRNA-24 binding and reducing its degradation (Yuan et al., 2017). Moreover, lncRNAs influence protein translation by recruiting translation initiation factors. LncRNA GAS5 binds eukaryotic translation initiation factor 4E (eIF4E), inhibiting c-Myc protein translation and downregulating its expression (Hu et al., 2014). It is worth noting that lncRNAs also play a role in regulating protein stability. Notably, lncRNAs regulate protein stability. For instance, lncRNA UPAT enhances UHRF1 stability by inhibiting β-transducin repeat-containing protein (TrCP)-mediated ubiquitination (Taniue et al., 2016).

Noncoding RNA molecules, such as lncRNAs (Salmena et al., 2011), circular RNAs (circRNAs) (Qin et al., 2023), or other RNA transcripts containing specific miRNA response elements (MREs), can serve as ceRNAs or miRNA sponges that bind to miRNAs and sequester them from their target mRNAs, thus modulating gene expression at post-transcriptional level. Many well-known lncRNAs have been demonstrated to exert important ceRNA roles in cancer. For example, lncRNA H19 promotes the angiogenesis of glioma by acting as a sponge for miR-29a and upregulating the angiogenesis factor vasohibin-2 (Jia et al., 2016). LncRNA CCAT1 can promote the proliferation and metastasis of esophageal squamous cell carcinoma (ESCC) by sponging miR-7 to enhance the expression of homeobox gene B1 (Zhang E. et al., 2017). Moreover, DANCR decoys two miRNAs, miR-335-5p and miR-1972, to facilitate ROCK1-mediated proliferation and metastasis of OS via a ceRNA network (Wang et al., 2018b).

LncRNAs were initially assumed to be ncRNAs that could not encode proteins. Nevertheless, recent evidence suggests that some lncRNAs contain open reading frames capable of encoding peptides, thereby allowing them to exert biological effects through the proteins they encode. For instance, the 53-amino acid peptide encoded by long non-coding RNA HOXB-AS3 has the capacity to suppress colon cancer (Huang et al., 2017). These transcripts are no longer considered *bona fide* ncRNAs, yet they are still categorized as a specific class of lncRNAs as they may play dual roles as both non-coding RNA or protein-coding transcripts in cancer.

## 3 Role of lncRNAs in OS oncogenesis and progression

**FIGURE 2:**
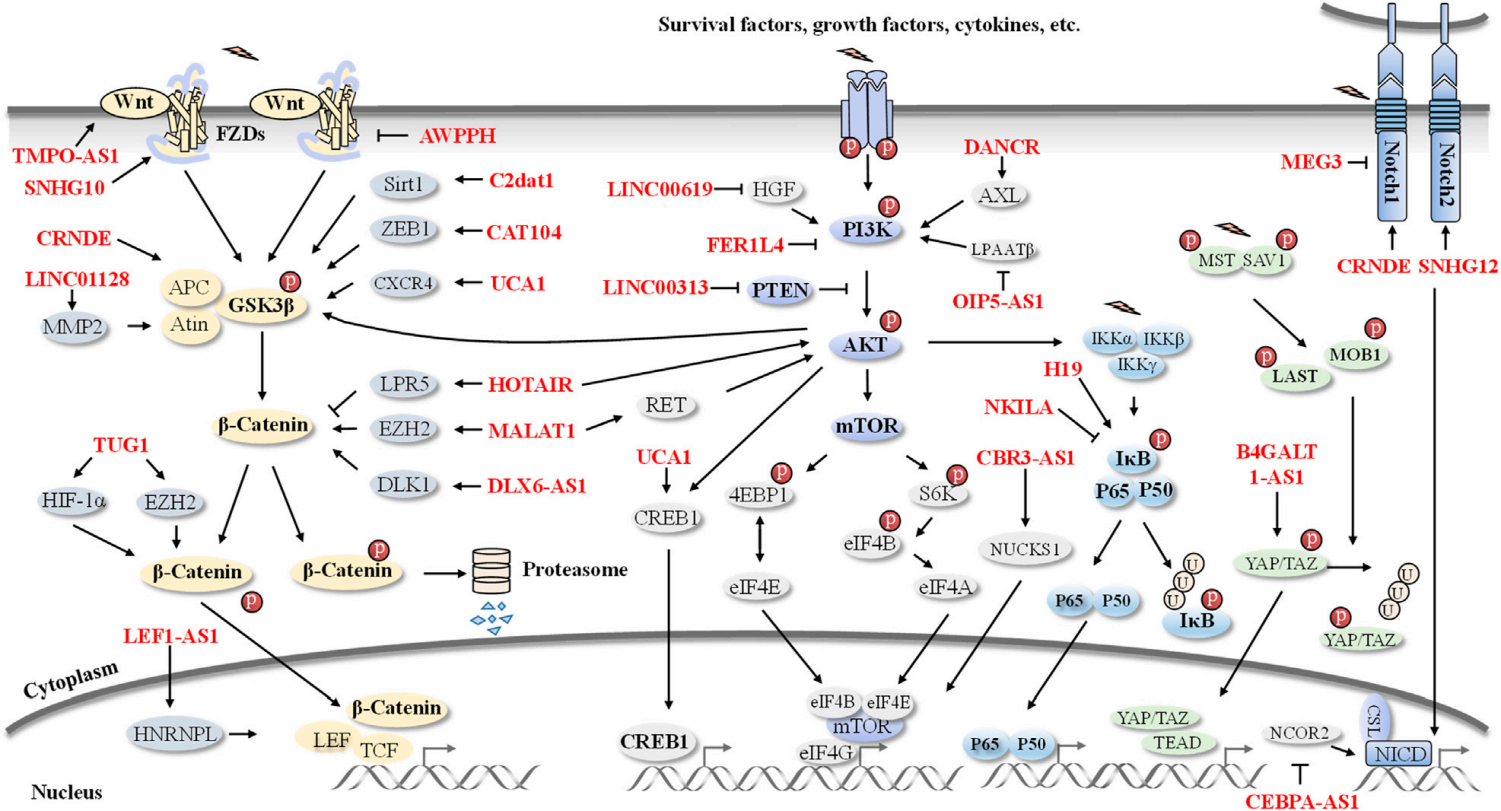
An overview of lncRNAs-involved signaling pathways in OS. Pathogenesis and progression of OS are regulated by several lncRNAs, including HOTAIR, MALAT1, UCA1, CRNDE, NKILA, CEBPA-AS1, H19, DANCR, and SNHG12 et al. These OS-related lncRNAs are involved in the Wnt/β-catenin, PI3K/AKT, NF-κB, Notch, and Hippo signaling pathways.

LncRNAs are involved in the regulation of cell proliferation, survival, metastasis, and invasion in OS via modulation of multiple oncogenic pathways, such as the Wnt/β-catenin, PI3K/AKT/mTOR, NF-κB, Notch, Hippo and HIF-1α pathways (Figure 2).

### 3.1 The Wnt/β-catenin pathway

The Wnt/β-catenin signaling pathway is a major player in the tumorgenesis of OS. This cascade is initiated by the binding of Wnt ligands to their receptors, Frizzled proteins, leading to the activation of Dishevelled (Dvl). Upon activation, Dvl triggers the phosphorylation of β-catenin, preventing its degradation by the proteasome and thus allowing its accumulation in the cytoplasm. Subsequently, β-catenin is translocated to the nucleus, where it binds to TCF/LEF transcription factors and induces the expression of target genes, including c-Myc and cyclin D1. This pathway can regulate processes such as proliferation, migration, apoptosis, and differentiation, while its dysregulation is associated with the development of various cancers, including OS (Nusse and Clevers, 2017).

#### 3.1.1 Regulation of proliferation

Several lncRNAs have been shown to control cell proliferation of OS via the Wnt/β-catenin signaling pathway. For instance, upregulation of LINC01128 facilitates cell proliferation in OS and exhibits a negative correlation with overall survival. *In vitro* and *in vivo* studies supported its role in modulating MMP2, a protease responsible for cleaving and releasing β-catenin from its cytoplasmic complex, by acting as a sponge of miR-299-3p, ultimately activating the Wnt/β-catenin signaling pathway (Yao and Chen, 2020). Similarly, lncRNA SNHG10 promotes OS growth by acting as a miR-182-5p sponge, which subsequently leads to increased FZD3 expression, thereby sustaining the activation of the Wnt/β-catenin signaling pathway (Zhu S. et al., 2020). Moreover, NF1A-AS1, overexpressed in OS, promotes cell proliferation by activating the Wnt/β-catenin signaling pathway, however, the underlying mechanism requires further exploration (Zhao et al., 2016). lncRNA LEF1-AS1 has been investigated to bind with HNRNPL and enhance the mRNA stability of LEF1, the nuclear effector of β-catenin, thereby promoting OS cell proliferation (Lu et al., 2020).

#### 3.1.2 Regulation of apoptosis

Wnt signaling has been proven to regulate apoptosis in OS, and recently, lncRNAs associated with Wnt signaling have also played an important role in regulating apoptosis (Brock et al., 2019). Depletion of TMPO-AS1 has been observed to restrain the Wnt/β-catenin pathway, thereby facilitating cell apoptosis (Cui and Zhao, 2020). CAT104 was highly expressed in OS cell lines and its knockdown significantly promoted OS-732 cell apoptosis. Mechanistically, CAT104 was found to regulate miR-381 to target ZEB1, as well as Wnt/β-catenin pathways, thus exerting its oncogenic effects on OS cells (Xia et al., 2018).

#### 3.1.3 Regulation of metastasis and invasion

Aggressive metastasis at early stages has been suggested to drive the rapid progression and poor prognosis of OS. Increasing evidence has pointed to the crucial role of Wnt cascade-associated lncRNAs in regulating the metastasis and invasion of OS. For instance, the knockdown of lncRNA CAMK2D-associated transcript 1 (C2dat1) reduced OS cell invasion and migration by modulating the miR-34a-5p/Sirt1 network and Wnt signaling pathway (Jia et al., 2018). Additionally, urothelial carcinoma associated 1 (UCA1) was reported to enhance cell viability, migration, and invasion by activating the Wnt/β-catenin signaling pathway (Zhu et al., 2018). EMT is an important process in which epithelial cells are transitioned into mesenchymal phenotype to gain invasive and metastatic capacities, thus worsening cancer patients’ survival rate (Pastushenko and Blanpain, 2019). CRNDE has been associated with the development of OS tumors, owing to its ability to trigger glycogen synthase kinase-3 (GSK-3) phosphorylation and consequent activation of Wnt/β-catenin target genes c-Myc and CCND1 expression (Ding et al., 2020). EZH2 is a key component of this pathway, acting as a negative regulator of Wnt/β-catenin signaling. EZH2 is known to interact with several components of the Wnt/β-catenin signaling pathway, including TCF/LEF transcription factors, β-catenin, and the co-activator CBP/p300, thus playing a crucial role in modulating their activity. EZH2 also interacts with other proteins involved in the Wnt/β-catenin pathway, such as GSK-3, which is involved in the phosphorylation and degradation of β-catenin. It was found that many lncRNAs can affect the Wnt/β-catenin pathway by regulating EZH2. For instance, MALAT1 can inhibit the expression of E-cadherin and promote the expression of β-catenin due to MALAT1-induced EZH2 activation (Zhao et al., 2022). Moreover, it has also been reported that TUG1 is involved in OS development, via mediating miRNA-144-3p/EZH2/Wnt/β-catenin pathway (Cao et al., 2017).

#### 3.1.4 Regulation of cell cycle

The dysregulation of the Wnt/β-catenin signaling pathway has been implicated in the disruption of cell cycle progression, which can lead to uncontrolled cellular proliferation (Ameratunga et al., 2019). Several studies have sought to uncover the association of lncRNAs with the Wnt signaling-driven cell growth in OS. For instance, BE503655 has been demonstrated to exert its effects on OS cell proliferation through regulation of the Wnt/β-catenin pathway. Silencing of BE503655 arrested OS cells in G0 and G1 phases and downregulated β-catenin and a range of Wnt cascades downstream targets, such as c-Myc, Cyclin D and MMP2(Huang et al., 2019). The lncRNA HOTTIP is the first lncRNA documented to regulate Wnt expression in OS. Its upregulation stimulates OS cell proliferation and cell cycle progression via activation of the Wnt/β-catenin pathway (Li Z. et al., 2016).

#### 3.1.5 Regulation of CSCs

Studies are increasingly suggesting a crucial role of lncRNAs in maintaining the stemness of CSCs (Shen et al., 2021). For instance, lncRNAs distal-less homeobox 6 antisense 1 (DLX6-AS1) was found to be highly expressed in OS tissue and cell lines. Moreover, its expression was positively correlated with tumor progression and metastasis, as well as poor prognosis. Mechanistically, DLX6-AS1 might act as a competing endogenous RNA for miR-129-5p to upregulate DLK1, consequently activating the Wnt pathway and promoting the stemness of OS CSCs (Zhang RM. et al., 2018).

### 3.2 The PI3K/AKT/mTOR pathway

The PI3K/AKT/mTOR pathway is a signaling pathway involved in the regulation of a variety of cellular processes such as growth, survival, metabolism, and motility in normal and cancer cells. This pathway is regulated by the phosphoinositide 3-kinase (PI3K) enzyme and its downstream effectors, the serine/threonine kinase AKT (also known as PKB) and the mechanistic target of rapamycin (mTOR) (Fruman et al., 2017). Researchers discovered that lncRNAs may exert their roles in OS evolution by targeting different components of this pathway. For example, HOTAIR silencing was found to inhibit the proliferation of MG-63 cells by inducing G1 phase arrest, which was associated with the decreased phosphorylation of mTOR and its upstream kinase AKT (Li E. et al., 2017). On the other hand, MALAT1 was found to be upregulated in both OS tissues and cell lines compared to normal controls, and its overexpression was associated with the proliferation, migration, and upregulation of stemness markers such as CD133, CD90 and SOX2 (Cai et al., 2016; Gao and Lian, 2016). Mechanistically, MALAT1 was found to bind competitively to miRNA-129-5p, blocking miRNA-129-5p-mediated degradation of RET, and thus activating the PI3K/AKT signaling pathway (Chen et al., 2018). LINC00313 has been reported to suppress PTEN expression, leading to AKT phosphorylation and promoting cell proliferation and metastasis *in vitro* and *in vivo* (Xing et al., 2022). Additionally, DANCR, an oncogenic lncRNA, has been found to promote the expression of the receptor tyrosine kinase AXL, activating the PI3K/AKT signaling pathway (Jiang et al., 2017). Furthermore, FER1L4 knockdown increases PI3K levels and AKT phosphorylation, consequently facilitating cell apoptosis and inhibiting EMT (Ye et al., 2019). Conversely, LINC00619 has been shown to inhibit the proliferation, migration, and invasion of OS cells by inactivating the HGF-mediated PI3K/AKT signaling pathway (Zi et al., 2022). Other lncRNAs such as UCA1(Ma et al., 2019), LINC00968(Liu et al., 2018), LINC00628(He et al., 2018), HULC (Kong and Wang, 2018), CCAT2 (Liu J. et al., 2019), PVT1 (Sun et al., 2019), LOXL1-AS1 (Chen et al., 2019), CBR3-AS1 (Yao et al., 2022) and DBH-AS1 (Liu ZB. et al., 2019) have also been implicated in the pathogenesis and progression of OS through the regulation of the PI3K/AKT signaling pathway.

### 3.3 The NF-κB pathway

Nuclear factor-κB (NF-κB) is a pivotal transcription factor that regulates cell growth, metastasis, and inflammation in cancer (Liu et al., 2015; Taniguchi and Karin, 2018). IκB (inhibitor of NF-κB), which binds to NF-κB in the cytoplasm and serves as a major brake to restraining NF-κB in an inactive form, is subject to phosphorylation by IκB kinases (IKK) after being activated by multiple stimuli, leading to its breakdown and eventual NF-κB activation (Taniguchi and Karin, 2018). Recently, a study concerning OS revealed that NKILA, a tumor suppressor that interferes with the NF-κB pathway in various cancer types, has lower expression levels in OS tissues and is associated with metastasis, tumor size, and the Enneking stage. Further investigation demonstrated that increased expression of NKILA suppresses the migration and invasion of OS cells by directly impeding the phosphorylation of IκB, thus inhibiting IKK phosphorylation and NF-κB activation (Liu M. et al., 2019). In addition, patients with high H19 expression had a shorter survival rate than those with low expression. H19 knockdown has been shown to reduce OS cell migration and invasion by affecting the levels of p-PI3K, p-AKT, and IκBα with no alteration in PI3K and AKT, suggesting that H19 knockdown interdicts migration and invasion of OS cells through blocking the NF-κB pathway (Zhao and Ma, 2018). Moreover, lncRNA NORAD (Xie et al., 2020) and XIST (Lu et al., 2017) have been reported to control cellular biological processes in OS by stimulating the NF-κB signaling pathway.

### 3.4 The Notch pathway

Notch signaling is known to be indispensable for development and is implicated in neoplastic transformation in multiple cancers. The Notch ligand binds to the Notch receptor on the surface of a contiguous target cell, thereby initiating the Notch signaling cascade. This cascade is then transmitted through the Notch effectors, the Notch-responsive transcription factors, and downstream target genes to determine cell fate through the regulation of cell proliferation, differentiation, and apoptosis processes (Radtke and Raj, 2003; Zhang et al., 2008; Takebe et al., 2014). Studies have demonstrated that the Notch pathway contributes to the pathogenesis of OS(Engin et al., 2009; Tao et al., 2014). The oncogenic role of CRNDE in OS is suggested to be mediated by regulating Notch1 signaling and EMT (Li et al., 2018b). Furthermore, SNHG12 promotes OS oncogenesis and metastasis by sponging miR-195-5p and consequently activating Notch2 (Zhou S. et al., 2018). On the contrary, tumor suppressor lncRNA MEG3 suppresses the proliferative and metastatic capabilities of MG63 and U2OS cells by reducing protein expressions of Jagged1, Notch1 and NICD1(Chen et al., 2020). Additionally, tumor suppressor lncRNA CEBPA-AS1 overexpression impedes OS progression by upregulating NCOR2 and attenuating Notch signaling pathway activation (Xia et al., 2020).

### 3.5 The Hippo signaling pathway

The Hippo signaling pathway is firmly established as a crucial regulator of tissue homeostasis and is implicated in the development of multiple human cancers, including OS (Badouel and McNeill, 2011; Morice et al., 2020). Activation of the Hippo kinase cascade leads to the phosphorylation of transcriptional co-activators YAP and TAZ, causing the retention of these proteins in the cytoplasm and subsequent degradation (Ji et al., 2017). Conversely, when the Hippo pathway is inactive, dephosphorylated YAP/TAZ are translocated to the nucleus, where they bind to the TEAD family and other transcription factors, thus stimulating gene transcription and consequently promoting tumorigenesis through cell proliferation and inhibition of apoptosis (Gumbiner and Kim, 2014). Recently, lncRNAs have been demonstrated to be involved in critical cancer-related signaling pathways, such as the Hippo signaling pathway. For instance, SOX2 and MYC, two downstream effectors of the Hippo pathway, are transcriptionally activated by ZMIZ1-AS1, resulting in augmented OS growth *in vivo* due to the stabilization of ZMIZ1 (Zhou et al., 2022). Moreover, silencing B4GALT1-AS1 impedes OS cell proliferation, migration, stemness, and chemotherapeutic sensitivity. Mechanistically, B4GALT1-AS1 recruits HuR to prolong YAP mRNA stability and thus its transcriptional activity (Li et al., 2018c). Additionally, LINC01089 impedes the progression of OS by activating the Hippo pathway (Zhang et al., 2022).

### 3.6 The HIF-1α pathway

As the most influential modulator in the reaction to hypoxia, hypoxia-inducible factor-1alpha (HIF-1α) has been corroborated to control hypoxic gene expression through diverse cellular components, including lncRNAs. LincRNA for kinase activation (LINK-A), an oncogenic activator of HIF1α signaling in breast cancer (Lin et al., 2016), displays a greater expression of plasma circulating LINK-A in metastatic OS patients in comparison to non-metastatic OS patients, demonstrating its potential role in OS metastasis. Augmented expression of LINK-A is shown to inhibit the expression of HIF1α. This inhibition can be reversed by treatment with the HIF inhibitor LW6, resulting in improved migration and invasion of OS cells. Hence, these results suggest that LINK-A might serve as an upstream activator of HIF1α to trigger the metastasis of OS (Zhao B. et al., 2019). TUG1 acts as a ceRNA to annul the endogenous effect of miR-143-5p, which repressed HIF1α expression to regulate OS cell metastasis (Yu et al., 2019).

### 3.7 Other signaling pathways

In addition to the signaling pathways already elucidated, some pathways are also correlated with lncRNAs in OS. The Mitogen-activated protein kinase (MAPK) signaling pathway is a pivotal modulator of cellular processes. Zinc finger antisense 1 (ZFAS1) has been reported to upregulate OS cells and promote colony formation, migration, and invasion through the activation of the MAPK signaling pathway (Liu CW. et al., 2019). Moreover, lncRNA H19, a key regulator of OS pathogenesis, is regulated by Hedgehog signaling and Yap1 overexpression (Chan et al., 2014). Additionally, LINC01116 is identified to upregulate IL6R in OS via targeting miR-520a-3p and stimulating the JAK/STAT signaling pathway (Zhang B. et al., 2018). With ongoing research, we may gain a better understanding of the significance of lncRNAs in OS pathogenesis by exploring their correlation with various cellular signaling pathways.

## 4 LncRNAs in OS drug resistance

**FIGURE 3:**
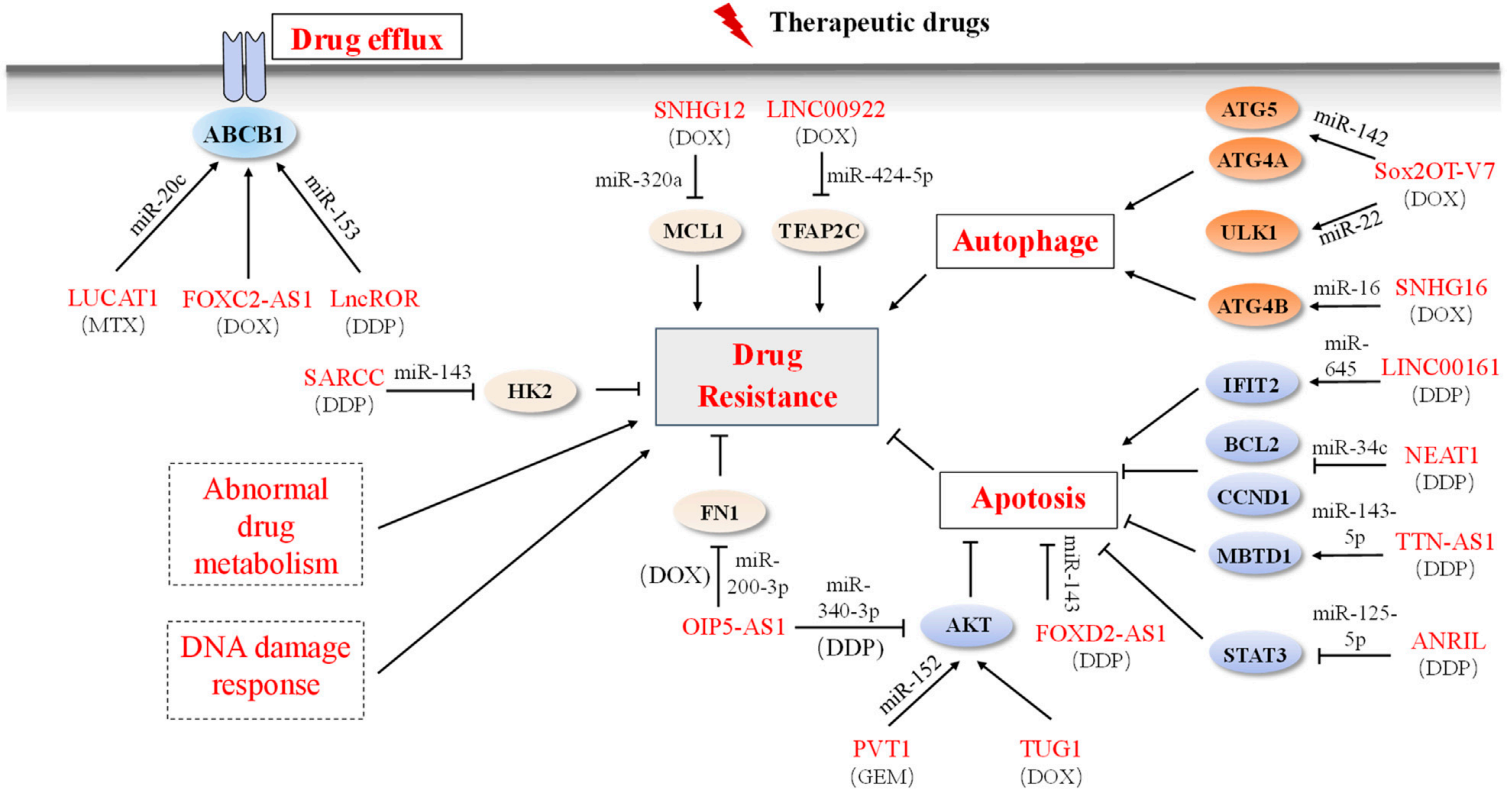
LncRNAs and mechanisms of drug resistance in OS. Dysregulated lncRNAs are involved in OS drug resistance by directly binding to related proteins or sponging miRNAs to alter the expression of downstream target genes involved in the drug efflux system, apoptosis, autophagy, and drug resistance-related signaling pathways. Solid boxes indicate the reported mechanism and dashed boxes indicate no OS-related lncRNAs participation mechanism.

The management of OS usually involves neoadjuvant chemotherapy, surgical excision, and post-operative adjuvant chemotherapy. Nevertheless, the effectiveness of this traditional approach is often impeded by drug resistance, which encompasses both primary and acquired resistance (Holohan et al., 2013; Housman et al., 2014). Primary resistance is innate, whereas acquired resistance is a result of the adaptability of cancer cells, and is driven by various aspects such as drug efflux, aberrant drug metabolism, autophagy, apoptosis, and upregulation of MDR-associated genes (Holohan et al., 2013; Leary et al., 2018). Common chemotherapeutic drugs used include cisplatin, doxorubicin, methotrexate, and gemcitabine (GEM) (Isakoff et al., 2015; Zheng et al., 2018). Of these, the emergence of secondary resistance to DOX and DDP is particularly concerning. Recently, several ncRNAs have been identified with the potential to impact chemosensitivity or chemoresistance in OS (Figure 3).

### 4.1 Drug efflux

The ATP-binding cassette (ABC) transporter family of proteins, which is implicated in ATP-dependent drug efflux, is essential for the maintenance of drug concentrations and successful treatments (Kathawala et al., 2015; Serra et al., 2021). However, augmented outflow and diminished inflow of drugs can have an antithetical effect, resulting in treatment failure (Gottesman et al., 2002; Belisario et al., 2020). LncRNAs have been demonstrated to modulate the expression of ABCs, conferring resistance in tumor cells (Ma et al., 2016). For example, lncRNA LUCAT1 has also been shown to modulate the classical drug resistance-related ABC subfamily B, member 1 (ABCB1) via sponging of miR-200c, leading to methotrexate resistance in OS (Han and Shi, 2018). Additionally, FOXC2-AS1 and its antisense transcript FOXC2 are observed to be highly expressed in DOX-resistant OS cell lines and tissues, predicting a poor prognosis. FOXC2 promotes chemotherapy resistance by inducing ABCB1 (Zhang CL. et al., 2017). The lncRNA ROR (regulator of reprogramming), markedly elevated in MG63/DDP and U2OS/DDP cells, as well as in relapsed OS tissues, is implicated in DDP resistance in OS through the miR-153/ABCB1 axis (Cheng et al., 2019).

### 4.2 Apoptosis

Apoptosis is a process by which a cell undergoes programmed cell death. There are two pathways by which this occurs: an intrinsic pathway, which is regulated by proteins of the BCL-2 family, caspase-9, and AKT, and an extrinsic pathway, which is mediated by death receptors on the cell surface. Ultimately, the intrinsic and extrinsic pathways converge and activate caspase-3, resulting in apoptosis (Elmore, 2007). Apoptosis resistance is a key factor in cancer cells’ ability to evade the effects of chemotherapeutic treatment (Song et al., 2009). LncRNAs have been demonstrated to modulate drug resistance by regulating apoptosis. For example, LINC00161 is induced by DDP and upregulates IFIT2, reversing the DDP-resistant phenotype of OS cells. Mechanistic studies show that LINC00161 sponges endogenous miR-645, leading to an increase in IFIT2 expression and enhanced DDP-induced apoptosis (Wang et al., 2016). On the contrary, NEAT1 has been observed to reduce sensitivity to DDP and impede DDP-induced apoptosis and cell cycle arrest, possibly by targeting miR-34c and disabling anti-apoptotic signals of the BCL-2 pathway and cell cycle-related cyclin D1 (CCND1) signals (Hu Y. et al., 2018). Similarly, LncRNA ANRIL, upregulated in OS, has been found to confer DDP resistance to U2OS and Saos-2 cells by targeting miR-125a-5p/STAT3 (Li and Zhu, 2019), while ANRIL knockdown significantly enhances DDP-induced cell apoptosis (Lee et al., 2021). LncRNA TTN-AS1 has been reported to increase the expression of MBTD1 by targeting miR-134-5p, thereby regulating cell growth, apoptosis, and drug resistance in OS (Fu et al., 2019). Furthermore, knockdown of lncRNA FOXD2-AS1 attenuates drug resistance, stimulates apoptosis, and weakens the invasion and migration capabilities of OS cells, plausibly due to the inhibition of miR-143 expression in drug-resistant cell lines (Zhang et al., 2021). Moreover, overexpression of lncRNA TUG1, a natural anti-tumor agent, is discovered in DOX-resistant OS cells, while downregulation of TUG1 by polydatincan promotes DOX-induced apoptosis *in vitro* and inhibits tumor growth in the DOX-resistant model *in vivo* by suppressing the AKT signaling pathway (Zhou Q. et al., 2020). Additionally, OIP5-AS1 inhibits apoptosis and stimulates various types of drug resistance by sponging different miRNAs. For example, OIP5-AS1 induces DDP resistance in OS by activating the LPAATβ/PI3K/AKT/mTOR signaling pathway through sponging miR-340-5p (Song et al., 2019). Another study reveals that OIP5-AS1confers DOX resistance in OS by upregulating fibronectin 1 (FN1) expression via sponging miR-200b-3p. This effect is reversed by inhibition of FN1, enhancing the sensitivity of OS cells to DOX both *in vitro* and *in vivo* (Kun-Peng et al., 2019). Moreover, lncRNA PVT1 plays a contributory role in the GEM resistance of OS cells through c-MET/PI3K/AKT pathway activation, which is largely dependent on miR-152 (Sun et al., 2019).

### 4.3 Autophagy

Autophagy is a lysosome-mediated process of intracellular degradation and recycling. It has been linked to drug resistance in cancer cells as it degrades pro-apoptotic proteins that might otherwise induce apoptosis. Autophagy can also increase cancer cell survival in response to drug treatments by degrading potentially harmful substances (Li YJ. et al., 2017). LncRNAs regulate autophagy and play an important role in drug resistance. For example, lncRNA Sox2OT-V7 contributes to the chemoresistance of OS to DOX (Wang W. et al., 2018). The underlying mechanism is that Sox2OT-V7 directly targets tumor-suppressive miR142/miR-22 to inhibit their expression, leading to the inhibition of downstream core ATG genes and consequently promotes DOX-induced autophagy and chemoresistance in OS cells (Zhu K. et al., 2020). Similarly, SNHG16 confers resistance to DDP in OS Saos-2 cells by upregulating ATG4B via miR-16, which induces autophagy (Liu Y. et al., 2019).

### 4.4 Abnormal drug metabolism

Abnormal drug metabolism is another potential mechanism of drug resistance in OS; lncRNAs can affect the expression of key metabolic enzymes, such as the cytochrome P450 (CYP) system, glutathione-S-transferase (GST) superfamily, and uridine diphosphate glucuronosyltransferase (UGT) superfamily, which play a role in the activation and inactivation of drugs (Michael and Doherty, 2005). Notably, tumor cells can increase their resistance to drugs by reducing their activation, as many anticancer drugs require metabolic activation (Housman et al., 2014). However, research into chemoresistance in OS has largely focused on drug efflux and cell death induction; the interplay between lncRNAs and drug-metabolizing enzymes has yet to be elucidated.

### 4.5 DNA damage response

The DNA damage response (DDR) is activated when cellular DNA is exposed to physical, chemical or biological factors, thus ensuring chromosomal stability (Jackson and Bartek, 2009). Chemotherapeutic agents are capable of inducing DNA damage and cytotoxicity; when repairable, this damage may either enable tumor cells to survive or become more resilient to chemotherapeutic agents. To our knowledge, chemotherapeutic drugs generate either direct (DDP) or indirect (DOX) DNA damage. Previous studies have revealed an intricate interplay between DDR genes and lncRNAs in cancer (Liu et al., 2013; Sharma et al., 2015). However, the association between lncRNAs and DDR in OS remains unexplored.

### 4.6 Other signal pathways involved

Apart from mediating drug resistance in OS via the mechanisms mentioned above, some lncRNAs have been identified as involved in other genes associated with drug resistance. For instance, LncRNA NORAD has been shown to inhibit the proliferation of OS HOS/DDP cells by targeting miR-410-3p, increasing their sensitivity to DDP (Xie et al., 2020). Furthermore, studies in both *in vivo* and *in vitro* settings have confirmed the role of lncRNA SARCC in enhancing OS DDP sensitivity. It can inhibit glycolysis by regulating Hexokinase 2 (HK2), a direct target of miR-143 in OS(Wen et al., 2020). Additionally, LINC00922 and SNHG12 have been revealed to contribute to OS DOX resistance via the miR-424-5p/TFAP2C axis and miR-320a/MCL1 axis respectively (Zhou B. et al., 2018; Gu et al., 2020). Nonetheless, further research is required to elucidate the exact mechanisms by which these lncRNAs are involved in drug resistance in OS and to explore ways to target them therapeutically.

Aberrantly expressed lncRNAs in OS show promising ability to predict chemotherapy response. Consequently, dynamic monitoring of these lncRNA levels may be a useful strategy for accurately assessing chemotherapy response in OS patients. Simultaneously, altering the expression of pertinent lncRNAs may be a strategy for reversing chemoresistance. In summary, combinatorial targeting of lncRNAs and conventional chemotherapy offers a novel approach for overcoming chemoresistance in OS, allowing for the selection of the best regimens and increased the efficacy of chemotherapy.

### 4.7 Regulatory network of lncRNA-miRNA-mRNA in OS

Compelling evidence highlights the regulatory network of lncRNA-miRNA-mRNA in OS. This competition for miRNA binding can influence the expression of genes involved not only in drug resistance but also in OS progression and outcome. Fascinatingly, it is noteworthy that a singular lncRNA can manifest diverse roles through its interactions with distinct miRNAs. For instance, the lncRNA PVT1 augments the chemoresistance of OS to GEM via its interaction with miR-152 (Sun et al., 2019). Simultaneously, PVT1 engages in miR-183-5p/ERG axis to propel the proliferation and metastasis of OS (Zhao W. et al., 2019). Likewise, the lncRNA NEAT1 has been observed to attenuate the sensitivity of OS cells to DDP and hinder DDP-induced apoptosis by targeting miR-34c and its downstream BCL-2 and CCND1 signals (Hu Y. et al., 2018). Additionally, NEAT1 facilitates the EMT and metastasis of osteosarcoma cells by sequestering miR-483, thereby upregulating STAT3 expression (Chen et al., 2021). Moreover, DANCR is engaged in miR-33a-5p/AXL (Jiang et al., 2017), miR-335-5p and miR-1972/ROCK1 axis (Wang et al., 2018b), thereby modulating OS progression through different signaling pathways. Given the substantial length of lncRNAs, it is plausible that distinct lncRNAs may share interaction sites with a particular miRNA. For instance, both GAS5 (Wang et al., 2021) and PINT (Liu, 2019) have shown the capacity to interact with miR-21, thereby exerting their tumor-suppressive roles.

It is worth highlighting that the utilization of a dual luciferase reporter assay and a RIP assay furnishes tangible evidence that fortify the ceRNA hypothesis. Nevertheless, the precise mechanistic model of ceRNA remains a subject of contention, with varying conclusions due to the abundance and affinity of these intracellular RNAs in ceRNA crosstalk. Several experimental investigations have unveiled the interplay between some lncRNAs and their ability to either foster or restrain tumorigenesis through distinctive ceRNA regulatory axes and subsequent downstream pathways. Further investigations are much warranted to unveil the intricacies inherent in lncRNA-miRNA-mRNA and/or lncRNA-protein networks. These captivating regulatory axes orchestrated by lncRNAs hold immense promise as novel therapeutic targets for OS. Here, we briefly describe the expression of some representative lncRNAs and their roles in OS (Table 1).

**TABLE 1:** Representative lncRNAs and their roles in OS.

LncRNA	Expression	Roles	Targets or effectors	References
A1BG-AS1	↑	Promotes proliferation	miR-148a-3p/USP22	Han et al. (2023)
AWPPH	↑	Promotes proliferation, migration and invasion	miR-93-3p/FZD7	Li et al. (2019)
CCAT2	↑	Promotes proliferation, migration and invasion	miR-200b/VEGF	Liu et al. (2019b)
CEBPA-AS1	↓	Inhibits proliferation and migration	miR-10b-5p/NCOR2	Xia et al. (2020)
DANCR	↑	Promotes proliferation and metastasis	miR-33a-5p/AXL; miR-335-5p and miR-1972/ROCK1	Jiang et al. (2017), Wang et al. (2018b)
DLGAP1-AS2	↑	Promotes progression and aerobic glycolysis	miR-451a/HK2	Zheng et al. (2023)
DLX6-AS1	↑	Promotes proliferation and metastasis	miR-641/HOXA9	Zhang et al. (2019b)
FAL1	↑	Promotes EMT	p21 and p-GSK-3β	Wang et al. (2018c)
FER1L4	↓	Promotes apoptosis and suppresses EMT	miR-18a-5p/SOCS5	Ye et al. (2019)
GAS5	↓	Inhibits EMT and lung metastasis	miR-21	Wang et al. (2021)
HOTTIP	↑	Promotes proliferation, migration and invasion	PTBP1/KHSRP	Yao et al. (2021)
HOTAIR	↑	Promotes proliferation and metastasis	LPR5; AKT1; MMP2 and MMP9; miR-217/ZEB1	Wang et al. (2015a), Li et al. (2017a), Wang et al. (2019), Li et al. (2023a)
H19	↑	Promotes migration and invasion	Yap1	Chan et al. (2014)
LINC00460	↑	Promotes proliferation and metastasis	miR-1224-5p/FADS1	Lian et al. (2019)
LINC00968	↑	Promotes proliferation and migration	MMP2 and MMP9	Liu et al. (2018)
LINC01123	↑	Promotes proliferation and metastasis	miR-516b-5p/Gli1	Pan et al. (2021)
LINK-A	↑	Promotes metastasis	HIF1α	Zhao et al. (2019a)
MALAT1	↑	Promotes angiogenesis and metastasis	miR-129-5p/RET; miR-150-5p/VEGFA; miR-509/Rac1	Chen et al. (2018), Zhang et al. (2018c), Vimalraj et al. (2021)
MEG3	↓	Inhibits Promotes migration and invasion	miR-361-5p/FoxM1; miR-21-5p/p53	Shen et al. (2019), Huang et al. (2023)
MIAT	↑	Promotes proliferation, migration and invasion, inhibits apoptosis	miR-128-3p/VEGFC	Zhang et al. (2019a)
NEAT1	↑	Promotes EMT and metastasis	miR-483/STAT3; miR-34c/BCL-2 and CCND1	Hu et al. (2018b), Chen et al. (2021)
PINT	↓	Inhibits proliferation, migration and invasion	miR-21	Liu (2019)
PVT1	↑	Promotes proliferation and metastasis, enhances chemoresistance	miR-183-5p/ERG; miR-152	Zhao et al. (2019b), Sun et al. (2019)
SNHG5	↑	Promotes proliferation and metastasis	miR-212-3p/SGK3	Ju et al. (2018)
SNHG12	↑	Promotes proliferation and metastasis, enhances chemoresistance	miR-195-5p/Notch2; miR-320a/MCL1	Zhou et al. (2018a), Zhou et al. (2018c)
SNHG14	↑	Enhances chemoresistance	miR-206/SLC7A11	Li et al. (2023b)
UCA1	↑	Promotes EMT and metastasis	miR-582/CREB1	Ma et al. (2019)
ZEB1-AS1	↑	Promotes proliferation and migration	ZEB1	Liu and Lin (2016)

## 5 Potential application of lncRNAs in OS

The current diagnosis of OS heavily relies on imaging tests, including X-rays, CT, MRI, and PET scans, which provide information on the tumor’s size, location, and spread within the body (Meyer et al., 2008; Strauss et al., 2021). However, despite the molecular features that identify patients at higher risk, such as RB1 loss, MYC amplification, and VEGFA amplification, none of these factors have been validated sufficiently to serve as a basis for risk stratification in clinical settings. Furthermore, the lack of reliable biomarkers and limited therapeutic options contributes to the high mortality rate of OS. Surgical resection remains the only possible curative approach for early OS, while patients with unresectable or widely metastatic OS are deemed incurable. Therefore, there is an urgent need to identify novel biomarkers and therapeutic targets for the improved diagnosis and treatment of OS.

### 5.1 LncRNAs as diagnostic biomarkers in OS

As previously discussed, many lncRNAs are aberrantly expressed in OS compared with normal tissue, which are useful to distinguish OS patients from healthy cohorts. Several studies reveal that the levels of OS-related lncRNAs can affect the activity, proliferation, and differentiation of osteoblasts, thereby directly reflecting the pathological staging and prognosis of patients. So far, Enneking staging has become an important basis for surgical staging of OS and is a better indicator of prognosis, aiding in the selection of appropriate treatment (Jawad and Scully, 2010). Various lncRNAs are associated with the clinical stage of Enneking, with higher malignancy correlating with higher stages and increased risk of distant metastasis. For instance, GAPLINC expression is increased in OS tissues and cell lines and is associated with poor histological grade, distant metastasis, and advanced Enneking stage. Increased GAPLINC expression is found to be an independent risk factor and inversely correlated with overall survival (Liao et al., 2018). Similarly, lncRNA GNAS-AS1 significantly increase in OS cells and tissues, which were positively correlated with the Enneking stage and distant metastasis (Mi et al., 2021). Moreover, lncRNA TUG1 (Yu et al., 2019), HOTAIR (Wang B. et al., 2015), DLX6-AS1 (Zhang N. et al., 2019) and HOTTIP (Li et al., 2015) are prominently increased in OS, which are associated with tumor size, distant metastases, TNM stage, overall survival and recurrence-free survival. These lncRNA biomarkers may provide a valuable method for the diagnosis and clinical staging of OS, offering a useful complement to existing biomarkers for more accurate clinical guidance.

Liquid biopsy is significantly less invasive than sampling solid tumor tissues; as a result, it makes an excellent diagnostic strategy. Intriguingly, several lncRNAs are identified as being cancer-specific and detected in body fluids (whole blood, plasma, urine, gastric juice, and saliva), which makes them promising biomarkers for cancer diagnosis and prognosis (Qi et al., 2016). The ability of lncRNAs to be released from cells and found encapsulated in exosomes in the extracellular space and bodily fluids is one of the confirmed methods. Recent research demonstrates that lncRNAs can be found in exosomes that have been extracted from the bodily fluids of cancer patients with a variety of malignancies (Li S. et al., 2018; Zhou R. et al., 2018; Li and Wang, 2021). As an alternative to traditional biomarkers, lncRNA-based tests in the circulatory system can be considered. Some plasma lncRNAs are linked to the growth and progression of tumors and may act as non-invasive circulating biomarkers for the detection of OS. Their classification, length, cellular localization, and mechanism of action have been discussed in another review (Moonmuang et al., 2021). For instance, UCA1 expression is much higher in OS tissues than in normal bone tissues, and it was also significantly higher in the serum of patients with OS than in the serum of healthy controls. Additionally, the tumor status, metastasis, and worse prognosis were all associated with its overexpression (Wen et al., 2017). Receiving operating characteristic (ROC) curve study shows that HNF1A-AS1 may distinguish patients with OS from healthy people. Upregulation of lncRNA HNF1A-S1 in OS serum is related to patients’ status (Cai et al., 2017). FAL1 may serve as a potential diagnostic marker for OS because it has recently been discovered that it is elevated in OS and that its serum level is related to the condition of the disease (Wang et al., 2018c). The low degree of invasiveness, affordability and timesaving procedures make a few circulating lncRNAs, whose expression coincides with the clinical stage of the disease, promising novel biomarkers to be added to current clinical practices for the management of OS.

### 5.2 LncRNAs as prognostic biomarkers in OS

Pulmonary metastasis and drug resistance are the two main factors that adversely affect overall survival prognosis. To improve existing treatment plans and establish the optimal timing for chemotherapy and surgery, the connection between lncRNAs biomarkers and OS prognosis must be explored. It has been found that lncRNAs have significant effects on cancer metastasis and medication sensitivity. Research shows that lncRNAs have a substantial impact on cancer metastasis and medication sensitivity. For instance, LncRNA BC050642 is found to be considerably more abundant in OS tissues and cell lines, and it was also proposed as an independent biomarker of OS prognosis (Yang Y. et al., 2019). The highly elevated LINC01614 in OS tissues predicts a poorer prognosis for patients. Knocking down LINC01614 inhibits OS cell proliferation, invasion, and metastasis. The miR-520a-3p/SNX3 regulatory axis, considered a potential clinical prognostic indicator for OS (Cai et al., 2021), is responsible for this effect. Additionally, TTN-AS1 may act as a carcinogenic agent by disrupting the miR-134-5p/MBTD1 gene and, given its high expression, can serve as a biomarker correlated with a poor prognosis (Fu et al., 2019). Other lncRNAs, such as FRFR-AS1 (Sun et al., 2016), BCAR4 (Wei et al., 2019), ZEB-AS1 (Liu and Lin, 2016), MALAT1 (Liu M. et al., 2019), DICER1-AS1 (Hu XH. et al., 2018), EWSAT1 (Zhang GY. et al., 2017), SOX2-OT (Wang et al., 2017) CCAL (Zhou et al., 2017), ATB (Han et al., 2017), and MEG3 (Shen et al., 2019), have been identified as potential prognostic biomarkers of OS. Although some lncRNAs have been pinpointed as prognostic markers of OS, their functions have yet to be validated and implemented in clinical practice. Thus, it is essential to identify lncRNA biomarkers for the prognosis of OS, particularly for patients with metastasis, to enable early diagnosis and potential therapeutic targets.

### 5.3 LncRNAs as promising OS therapeutic targets

Currently, the standard treatment for OS is surgery to remove the tumor followed by chemotherapy. Targeted drugs are being developed to treat OS, but none have been approved yet (Martin Broto et al., 2022). Chemoresistance resistance is frequently observed during OS treatment. Therefore, the prognosis of OS has not been improved for a long time, which is at least in part due to a lack of therapeutic target and chemoresistance. Thus, a novel target is required for improving the prognosis of OS patients. LncRNAs have emerged as attractive candidates due to their cell-, tissue-, and disease-specific expression patterns, and pre-clinical studies have demonstrated promising results in the treatment of disorders, including cancer (Arun et al., 2016; Papaioannou et al., 2019). Moreover, lncRNA-targeting approaches have advantages over protein-targeting methods, as the base-pairing principle is simpler than designing a specific protein-binding inhibitor. Targeting lncRNAs can be achieved using small interfering RNA (siRNA), antisense oligonucleotides (ASOs), ribozymes, aptamers, and miRNAs, which have all been evaluated for targeting cancer-associated genes (Parasramka et al., 2016; Pierce et al., 2022). For instance, Arun et al. administered subcutaneously injected mice with 16-mer gapmer targeting MALAT1 modified with alternative cEt-constrained nucleotides and phosphorothioate bonds before the spontaneous development of mammary tumors, resulting in a 50% reduction in metastatic burden, and a highly differentiated, cystic/ductular histological phenotype resembling MALAT1^−/−^ genomic knockout mice (Arun et al., 2016). Similarly, patient-derived xenograft (PDX) mouse models demonstrated that the treatment with lipid nanoparticle (LNP)-formulated anti-HOXB-AS3 gapmers effectively downregulated HOXB-AS3 expression in human CD45-selected AML blasts isolated from the bone marrow of the treated mice. This treatment significantly prolonged the survival of the treated mice compared to the control group, with the median survival of the scramble-treated mice being only 63 days, whereas it was not reached after 100 days of treatment for the HOXB-AS3 KD group (Papaioannou et al., 2019).

Drug resistance-associated lncRNAs, such as LUCAT1(Han and Shi, 2018), ANRIL (Lee et al., 2021), Sox2OT-V7 (Zhu K. et al., 2020) and others, may be useful as therapeutic targets to reverse chemotherapy resistance in the future. In addition, lncRNAs, such as LINC00588 (Zhou FC. et al., 2020), GAPLINC (Liao et al., 2018), HOTAIR (Wang B. et al., 2015) and DNACR (Wang et al., 2018b), which are up- or downregulated in OS and regulate OS metastasis, could be targets for therapeutic intervention. For instance, lncRNA LINC00588 is expressed differently in cancer in bone and metastatic carcinoma in the lung and may act as a ceRNA to affect the expression of TP53. Thus, the LINC00588/miRNA-1972/TP53 axis may serve as a potential therapeutic target in OS patients (Zhou FC. et al., 2020). Overall, targeting these lncRNAs could be used to reverse drug resistance or prevent OS metastasis. However, further clinical trials and transformation research are needed to determine which lncRNAs are key targets and to elucidate the mechanism of lncRNAs in OS drug resistance.

## 6 Conclusion

LncRNAs, a prominent topic in RNA biology and oncology, are involved in multiple aspects of OS progression and development. Recent studies have revealed that alterations in transcription or processing can lead to alterations in the expression of both oncogenic and tumor-suppressive lncRNAs. These lncRNAs bind to DNA, RNA, or proteins, affecting the expression, localization, stability, and activity of their binding partners, thus driving cancerous phenotypes such as persistent proliferation, metastasis, increased stemness, and drug resistance, resulting in tumorigenesis and OS progression. As a result, lncRNAs present in bodily fluids can be utilized as biomarkers for OS, offering high sensitivity, or in combination with other molecules to enhance clinical efficacy and accuracy. Further research into lncRNA roles and functions in OS may lead to novel strategies to modulate lncRNA expression and benefit OS treatment and management.

Functional studies of lncRNAs can be challenging due to their overall lower sequence conservation compared with protein-coding genes (Necsulea et al., 2014). Recent research has revealed that lncRNAs tend to maintain evolutionarily conserved secondary structures in the presence of considerable sequence variation (Juan et al., 2000). It has been suggested that selective pressure may act on lncRNAs at the secondary structure level to constrain sequence variation, resulting in reduced mutations in structurally critical regions (Pegueroles and Gabaldón, 2016). This contrasts with protein-coding genes, which are generally more highly conserved since even a single nucleotide insertion or deletion can cause a frameshift and complete loss of function. This implies that the secondary structure of lncRNAs may not only be an evolutionary constraint but also the functional unit involved in biological processes. However, the secondary structure of lncRNAs has not been sufficiently studied, nor has the relationship between lncRNAs secondary structure and its biological functions been revealed. Another study also points out that lncRNAs exhibit common motifs and motif combinations, conserved secondary structures, and small sequence elements that are key determinants of their function (Ross et al., 2021). Despite the progress made in understanding lncRNA structure-function relationships, much more research needs to be done to comprehend the structure and functionality of the thousands of lncRNAs. Moreover, a multitude of investigations have revealed the pivotal role of ncRNAs, particularly circRNAs (Qin et al., 2023; Zeng et al., 2023) and lncRNAs (Wang et al., 2020), as ceRNA in the regulation of malignant biological properties, encompassing cell proliferation, invasion, metastasis, and chemotherapy resistance in OS (Table 1). However, the direct experimental evidence elucidating the involvement of ceRNA remains insufficient, necessitating further exploration into the intricate mechanisms governing their regulation.

Another concern when studying lncRNAs is the absence of effective tools to manipulate their expression or function. Traditional methods for studying gene function, such as gene knockout or overexpression, are often not possible for lncRNAs due to their large size and complex structure. Additionally, lncRNAs often lack conserved functional domains or motifs, making it difficult to predict their function based on sequence alone. Thus, CRISPR/Cas9-mediated knockout (Yang et al., 2018) is often not suitable for the study of lncRNAs because lncRNAs have biological functions that are less likely to be perturbed by small indels. Moreover, RNA interference (RNAi) (Heale et al., 2005), and antisense oligonucleotide (ASO) (Meng et al., 2015) and Cas13-based methods (Zhang et al., 2020) for knockdown have a significant risk of false positives caused by off-target effects, and the introduction of exogenous molecules may be toxic to organisms. Meanwhile, CRISPR interference (CRISPRi) works by using a deactivated Cas9 (dCas9) protein fused to a transcriptional repressor domain to selectively silence the expression of a particular lncRNA without affecting neighboring genes or ncRNAs. The advantages of CRISPRi over other methods for modulating lncRNA expression include its specificity, reversibility, and the potential to reveal the function of lncRNAs. However, more accurate mapping of lncRNA TSSs, optimizations to the design of sgRNA sequences, and potential off-target effects are still needed.

Obtaining reliable clinical biomarkers for early diagnosis and treatment of OS is a key issue. Circulating lncRNAs may be more reliable cancer biomarkers than CTCs, cfDNA (including circulating tumor DNA, ctDNA) and exosomes due to their high stability and relative abundance. They may be used as a biomarker for early diagnosis of OS; however, challenges remain in establishing a real-time and minimally invasive monitoring system using circulating lncRNA signatures. The main challenge at this stage is the difficulty in meeting the demand for both sensitivity and specificity. Circulating lncRNAs may struggle with sensitivity and specificity when targeting specific cancer species for indicated detection. Thus, a combination of several blood-based lncRNAs for signature offers better diagnostic performance than most individual circulating lncRNAs. For example, a signature composed of three lncRNAs (PTENP1, LSINCT-5, and CUDR) significantly outperforms CEA and CA19-9 in gastric cancer diagnosis studies (Dong et al., 2015). Additionally, new technologies, such as dual electrochemical genosensor (Sánchez-Salcedo et al., 2021), can help advance circulating lncRNAs as tumor markers for clinical applications. Moreover, to improve feasibility as a biomarker for OS, larger and more diverse cohorts are also needed to confirm its diagnostic potential.
